# Nogo-C regulates cardiomyocyte apoptosis during mouse myocardial infarction

**DOI:** 10.1038/cddis.2016.331

**Published:** 2016-10-20

**Authors:** Shi Jia, Xue Qiao, Jingjing Ye, Xuan Fang, Chunling Xu, Yangpo Cao, Ming Zheng

**Affiliations:** 1 Department of Physiology and Pathophysiology, Health Science Center, Peking University, Beijing 100191, China

## Abstract

Myocardial infarction is caused by insufficient coronary blood supply, which leads to myocardial damage and eventually the heart failure. Molecular mechanisms associated with the loss of cardiomyocytes during myocardial infarction (MI) and ischemia-related cardiac diseases are not yet fully understood. Nogo-C is an endoplasmic reticulum protein ubiquitously expressed in tissues including in the heart, however, the cardiac function of Nogo-C is still unknown. In the present study, we found that Nogo-C was upregulated in mouse hearts after MI, and hypoxic treatments also increased Nogo-C protein level in cardiomyocytes. Adenovirus mediated overexpression of Nogo-C led to cardiomyocyte apoptosis, whereas knockdown of Nogo-c by shRNA protected cardiomyocytes from hypoxia-induced cell apoptosis. Importantly, Nogo-C knockout mice displayed improved cardiac function, smaller infarct area, and less apoptotic cells after MI. Moreover, we found that miR-182 negatively regulated Nogo-C expression and was downregulated during MI, expressing miR-182 in cardiomyocytes protected hypoxia- and Nogo-C-mediated cell apoptosis. Our results indicate that increased cardiac Nogo-C expression is both sufficient and necessary for ischemia-induced cardiomyocyte apoptosis and cardiac dysfunction, suggesting that deregulation of Nogo-C by miRNA may be a potential therapeutic target for ischemia-related heart diseases.

Myocardial infarction (MI) causes myocardial damage and eventually leads to heart failure, the leading cause of death worldwide.^1, 2^ MI is characterized by insufficient coronary blood supply, and the prolonged ischemia during MI causes loss of cardiomyocytes due to apoptosis and necrosis.^3^ Although various signaling pathways involved in cardiomyocyte apoptosis including reactive oxygen species (ROS), protein kinase C, MAPK, and nuclear factor kB, have been reported,^4, 5, 6, 7^ the molecular mechanisms associated with MI and ischemia-related cardiac diseases are not yet fully understood.

Neurite outgrowth inhibitor proteins (Nogo) belong to reticulon (RTN) protein family, which is characterized by the endoplasmic reticulum targeting motif at the carboxy terminal.^8^ Nogo gene encodes three splicing isoforms, Nogo-A, Nogo-B and Nogo-C. Nogo-A is a membrane protein expressed mainly in central nervous system such as oligodendrocytes and neurons, serving as a growth inhibitory factor and restricting axon re-extension.^9, 10^ Recently, Nogo-A has also been found as an important negative regulator of developmental angiogenesis in central nervous system.^11^ Nogo-B is a shorter isoform than Nogo-A, expressed ubiquitously in the body.^12^ In peripheral nervous system, Nogo-B is expressed in Schwann cells and the interaction of Nogo-B with its receptor on neurons mediates axonal branching, indicating a role in the excessive axonal sprouting after peripheral nerve injury.^13^ In peripheral blood vessels, Nogo-B regulates endothelial cell migration thus mediating vascular remodeling after lesions.^14^ And in pulmonary arterial hypertension patients, Nogo-B is increased and causes pulmonary arterial smooth muscle cell over-proliferation due to suppression of cell apoptosis.^15^ Despite these important functions from *in vitro* studies, either Nogo-A or Nogo-B knockout mouse displays relatively normal phenotypes.^16^ Nogo-C is the shortest protein in Nogo family, and expressed in many tissues, including liver, neuron, vascular smooth muscle cells, skeletal muscles, and heart.^17^ In transgenic mice expressing Nogo-C in Schwann cells, sciatic nerve injury causes delayed axonal regeneration and decreased recovery of motor function.^18^ In hepatic carcinoma, the protein level of Nogo-C is negatively correlated with tumor size and prognosis.^19, 20^ However, the role of Nogo-C protein in cardiac pathogenesis has not been investigated.

As Nogo proteins have been reported to mediate multiple cell apoptosis,^21, 22, 23^ and apoptosis is a common feature of cardiac infarction, we then hypothesize that Nogo-C may have a role in mediating cardiomyocyte apoptosis during cardiac infarction. In the present study, using combination of *in vitro* and *in vivo* approaches, we investigated the role of Nogo-C in MI and ischemic cardiomyocytes. We found upregulated protein level of Nogo-C in MI heart tissues and hypoxic cardiomyocytes. Nogo-C deletion preserved cardiac functions after MI. Our study provides *in vivo* evidence, for the first time, that Nogo-C has a pivotal role in regulating cardiac function and may serve as a potential therapeutic target for clinical MI treatment.

## Results

### Nogo-C expression was upregulated in MI heart and hypoxic cardiomyocytes

**Figure 1:**
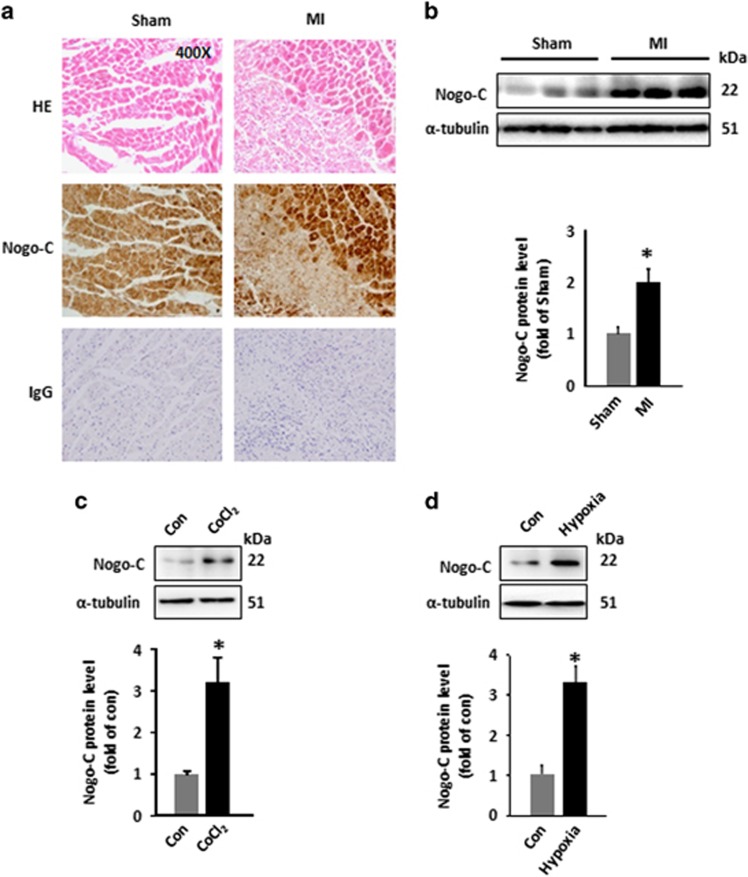
Nogo-C is upregulated in MI hearts and hypoxic cardiomyocytes. (**a**) Hematoxylin/eosin staining and Nogo-C immunohistochemical staining of sham and MI mouse hearts, IgG staining as control. (**b**) Western blot and average data showing Nogo-C protein levels in sham and MI mouse hearts 24 h after operation. *n*=6–7 mice. (**c**) and (**d**) Nogo-C protein levels in rat neonatal cardiomyocytes treated with CoCl_2_ (600 *μ*M) for 24 h (**c**) or with hypoxia incubation (1% O_2_) for 12 h (**d**). *n*=6 independent experiments.*P<0.05 *versus* sham group or control cells

Nogo-C expressed ubiquitously in mouse tissues including the heart as indicated by its expression pattern (data not shown). To understand the pathological role of Nogo-C in the heart, we checked the expression level in MI. Immunohistochemical staining by Nogo-C antibody showed that Nogo-C was upregulated in the border zone of infarct area 24 h after LAD (Figure 1a). Western blot result confirmed the increased Nogo-C protein level in the border zone of MI, as indicated by the 2.3-fold increase in MI mouse heart than in sham control (Figure 1b). We then tested if hypoxia promoted Nogo-C protein level in cultured neonatal cardiomyocytes by two approaches. First, we treated cardiomyocytes with CoCl_2_, a compound commonly used as a hypoxic inducer due to its role in inducing and stabilizing hypoxia inducible factor 1*α* (HIF-1a). CoCl_2_ (600 *μ*M for 24 h) caused increased Nogo-C protein level to 3.2-fold of that in vehicle control cells (Figure 1c). Then, we cultured cardiomyocytes in hypoxic incubator with gas containing 94% N_2_, 1%O_2_ and 5%CO_2_ for 12 h, and found that hypoxia treatment also increased Nogo-C to 3.3-fold comparing with cardiomyocytes cultured in normoxia condition (Figure 1d). Together, these results showed that Nogo-C protein was changed during hypoxia or MI, suggesting that Nogo-C may have a role in the pathogenesis of hypoxia-related cardiac diseases.

### Nogo-C induced cardiomyocyte apoptosis

**Figure 2:**
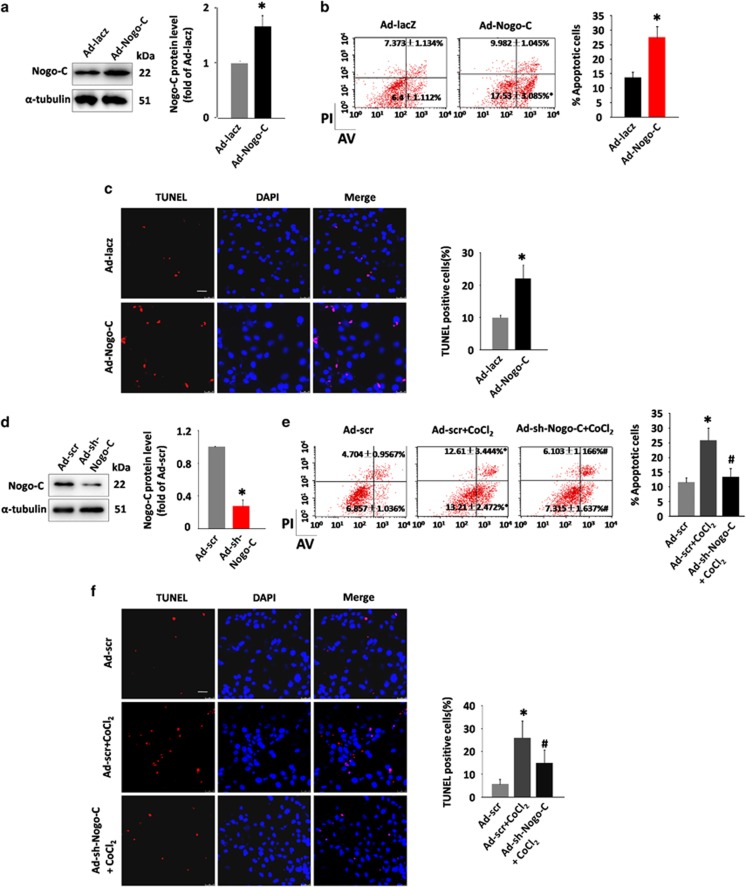
Nogo-C regulates cardiomyocyte apoptosis. (**a**) Western blot showing the Nogo-C protein level in rat neonatal cardiomyocytes transfected with Ad-Nogo-C at 50 MOI or Ad-laz for 48 h. *n*=3 independent experiments. (**b**) Flow cytometry analysis and (**c**) TUNEL staining showing apoptotic rates of rat neonatal cardiomyocyte transfected with Ad-Nogo-C or Ad-laz. *n*=3 independent experiments for flow cytometry analysis; and *n*=6 independent experiments for TUNEL staining. Scale bar=25 *μ*m. (**d**) Western blot showing the Nogo-C protein level in rat neonatal cardiomyocytes transfected with Ad-sh-Nogo-C at 50 MOI or Ad-scramble for 48 h. *n*=3 independent experiments. (**e**) Flow cytometry analysis and (**f**) TUNEL staining showing apoptotic rates of rat neonatal cardiomyocytes infected with Ad-sh-Nogo-C or Ad-scramble in response to CoCl_2_ stimulation (600 *μ*M). *n*=6 independent experiments. Scale bar=25 *μ*m. *P<0.05 *versus* scramble control cells. ^#^*P*<0.05 *versus* scramble+CoCl_2_ cells

We next investigated if alteration of Nogo-C protein level mediated cardiomyocyte function. Transfection of rat neonatal cardiomyoyctes with adenovirus containing Nogo-C cDNA (Ad-Nogo-C) caused increased cellular Nogo-C protein level (Figure 2a). Overexpression of Nogo-C increased cardiomyocyte apoptosis, as indicated by flow cytometry assay, with a LR (LR quadrant indicates the percentage of early apoptotic cells to Alexa 488 stained cells) of 17.53±3.085% in Ad-Nogo-C expressing cells and 6.4±1.112% in vector control cells, and a UR (UR quadrant indicates the percentage of late apoptotic cells to Alexa 488 and propidium iodide-stained cells) of 9.982±1.045% in Ad-Nogo-C cells and 7.373±1.134% in control (Figure 2b). The apoptotic effect of Nogo-C on cardiomyocytes was also confirmed by TUNEL staining, with a 2.2-fold increased apoptotic rate in adeno-Nogo-C cells over that in control cells (Figure 2c). Thus, our results suggest that Nogo-C is sufficient to induce cardiomyocyte apoptosis.

We then investigated if decreased Nogo-C protein level could protect cardiomyocytes from apoptotic stimuli by infecting cardiomyocyte with adenovirus containing Nogo-C short hairpin RNA (shRNA) (Ad-sh-Nogo-C) to knockdown Nogo-C protein (Figure 2d). Flow cytometry assay showed that Nogo-C knockdown protected cardiomyocyte apoptosis induced by CoCl_2_, from 12.61±3.444% to 6.103±1.166% (LR) and 13.21±2.472% to 7.315±1.637% (UR) (Figure 2e). Similarly, TUNEL staining showed that knockdown of Nogo-C decreased CoCl_2_ induced cardiomyocyte apoptosis from 25.8% to 13.3% (Figure 2f). Altogether, our results showed that Nogo-C is both sufficient and necessary for cardiomyocyte apoptosis.

### Knockout of Nogo-C protected mice from MI-induced cardiac dysfunction

**Figure 3:**
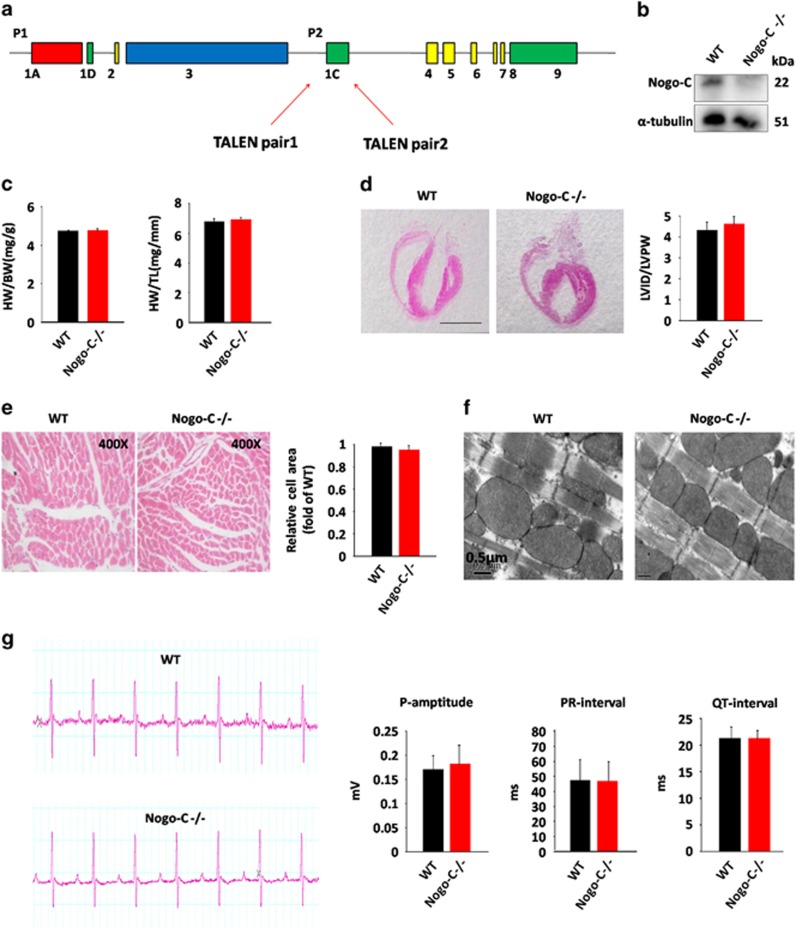
Generation and characterization of Nogo-C Knockout mouse. (**a**) Schematic diagram of Nogo-C^−/−^ mouse construction by TALEN technique. (**b**) Western blot showing the Nogo-C protein level in hearts from wildtype and Nogo-C^−/−^ mice. (**c**) Heart weight to body weight ratio (left) and Heart weight to tibia length ratio (HW/TL) (right) of 8-week male WT and Nogo-C^−/−^ mice. *n*=11 mice. (**d**) Hematoxylin and eosin staining showing the heart chambers of 8-week male WT and Nogo-C^−/−^ mice (left), and the diastole LVID to LVPW ratio (right). Scale bar=0.5 cm. *n*=6. (**e**) Cross section image of 8-week male WT and Nogo-C^−/−^ mouse hearts by hematoxylin and eosin staining (left) and the average data (right). WT, *n*=70 cells (from 4 mice); Nogo-C^−/−^, *n*=63 cells (from 5 mice). Magnifying power: 400X. (**f**) Representative TEM images of 8-week male WT and Nogo-C^−/−^ mouse hearts. Scale bar=0.5 *μ*m. (**g**) Electrocardiograph images of 8-week male WT and Nogo-C^−/−^ mice (left) and statistic data for P-amptitude, PR-interval, and QT-interval (right). *n*=4–5 mice

To understand the *in vivo* function of Nogo-C, we generated Nogo-C^−/−^ mice by TALEN technique to delete the Nogo-C specific exon 1c (Figures 3a and b). Basically, the ratios of heart weight to body weight and heart weight to tibia length in Nogo-C^−/−^ mice are similar with that in control mice at age of 8-week old (Figure 3c). Also, left ventricular internal diameter (LVID)/left ventricular posterior wall thickness (LVPW) showed no difference between Nogo-C^−/−^ and control mice (Figure 3d). Cardiomyocyte area by hematoxylin/eosin staining and ultra-structure by tansmission electron microscope are all similar in hearts of Nogo-C^−/−^ and control mice (Figures 3e and f). Moreover, Nogo-C knockout did not alter cardiac function as revealed by echocardiography (Table 1) and electrocardiograph assay (Figure 3g). These data suggest that deletion of Nogo-C does not affect heart morphology and function at basal conditions.

**Figure 4:**
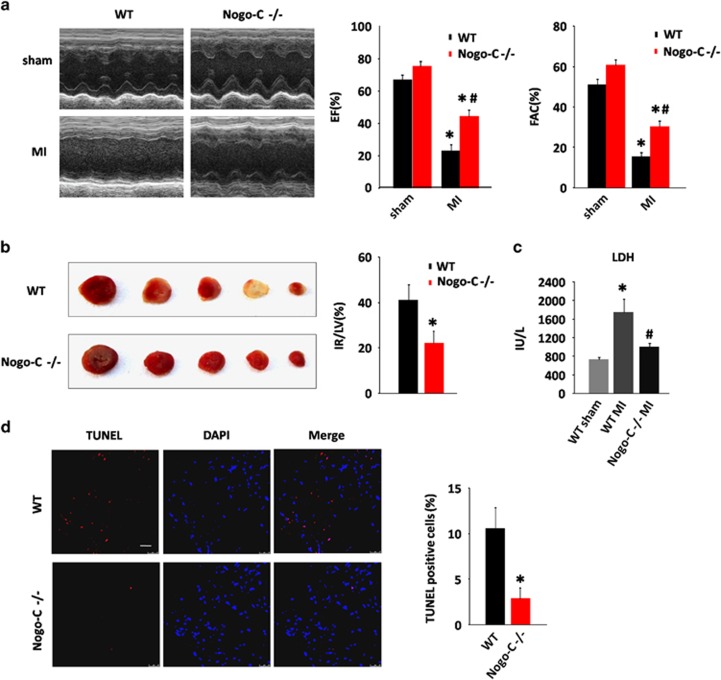
Nogo-C knockout protects mouse heart from MI injury. (**a**) Typical example of M-mode echocardiograms of wildtype and Nogo-C^−/−^ mouse hearts with (MI) or without (sham) LAD ligation for 24 h (left), and average data of EF (middle) and FAC (right). *n*=6 mice for each group. *P<0.05 *versus* sham wildtype mice; ^#^*P*<0.05 *versus* MI wildtype mice. (**b**) Representative triphenyltetrazolium chloride (TTC) staining images of sequential heart sections (left), and the average data of TTC infarction size (right) of WT and Nogo-C^−/−^ mice after LAD ligation for 24 h. *n*=4 mice,*P<0.05 *versus* wildtype mice. (**c**) Serum LDH activities of wildtype and Nogo-C^−/−^ mice with or without LAD ligation for 24 h. *n*=5–11 mice. *P<0.05 *versus* WT sham; ^#^*P*<0.05 *versus* WT MI. (**d**) TUNEL staining showing apoptotic cells in the border zone of MI from WT and Nogo-C^−/−^ mouse hearts 24 h after LAD ligation (left), and the average data (right). *n*=4 mice. Scale bar=25 *μ*m. *P<0.05 *versus* wildtype mice

We then investigated the possible effect of Nogo-C deficiency on cardiac function during MI. Although echocardiography analysis showed largely decreased ejection fraction (EF) and fractional area change (FAC) in control mice after MI (EF: 67.26±3.148% *versus* 23.51±3.694%, FAC: 50.9±2.992% *versus* 15.44 ±2.338%, before and after MI), Nogo-C knockout significantly improved cardiac function after MI to 44.61±4.164% (EF) and 30.49±2.77% (FAC) (Figure 4a). The infarct size (IR) of Nogo-C^−/−^ mouse hearts also decreased as comparing with wildtype littermates after MI (Figure 4b). Lactate dehydrogenase (LDH) levels in Nogo-C^−/−^ mouse serum after MI was reserved as comparing with the increased levels in control littermates after MI (1004±76.79 in Nogo-C^−/−^ mouse *versus* 1740±289.9 in control mouse after MI) (Figure 4c), indicating that knockout of Nogo-C has a protective effect on MI-induced heart injury. We further examined cardiomyocyte apoptosis by TUNEL staining after MI, and found that Nogo-C knockout protected cardiomyocyte apoptosis, from 10.64±2.255% in control hearts after MI to 2.907±1.163% in Nogo-C^−/−^ hearts (Figure 4d). Collectively, these data support the protective role of Nogo-C knockout on MI-induced heart damage.

### MiR-182 negatively regulated Nogo-C expression during MI

**Figure 5:**
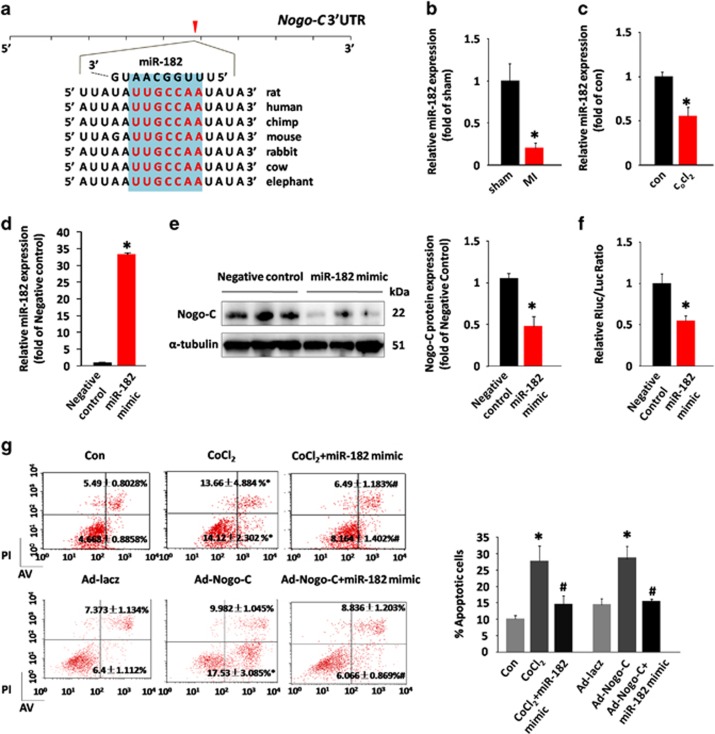
miR-182 regulates Nogo-C expression. (**a**) Analysis of Nogo-C 3′UTR region predicting miR-182 as a regulator. PCR data showing miR-182 expression in MI hearts (**b**) or CoCl_2_-treated cardiomyocytes (**c**). (**d**) Expression of miR-182 in control and miR-182 mimic transfected rat neonatal cardiomyocytes. (**e**) Western blot (left) and average data (right) showing the Nogo-C protein levels in control and miR-182 overexpressing rat neonatal cardiomyocytes. (**f**) Luciferase reporter activity of rat Nogo-C 3′ UTR in cells transfected with miR-182 mimic or scrambled miRNA mimic for 48 h. (**g**) Flow cytometry analysis showing apoptotic rates of rat neonatal cardiomyocytes transfected with or without miR-182 mimic in response to CoCl_2_ stimulation (600 *μ*M) or Nogo-C overexpression. *n*=6 independent experiments. *P<0.05 *versus* control cells. ^#^*P*<0.05 *versus* CoCl_2_ or Nogo-C overexpressing cells

MiRNAs have a critical role in cardiac pathological processes. In this study we tried to identify the possible miRNA that regulates Nogo-C gene expression during MI. *In silico* analysis predicted that miR-182 targets the 3′ untranslated region (3′UTR) of Nogo-C (Figure 5a). Importantly, miR-182 expression was significantly decreased in the heart during MI (Figure 5b) or in CoCl_2_-treated cardiomyocytes (Figure 5c). Overexpression of miR-182 dramatically reduced Nogo-C protein expression in cardiomyocytes (Figures 5d and e). In addition, miR-182 decreased Nogo-C 3′-UTR luciferase reporter activity (Figure 5f), further confirming that Nogo-C is a target gene of miRNA-182.

To test if miR-182 had a role in Nogo-C-induced cardiomyocyte apoptosis, cardiomyocytes were co-transfected with miR-182 and Ad-Nogo-C, or transfected with miR-182 in the presence of CoCl_2_. Although overexpression of Nogo-C or treatment of CoCl_2_ induced cardiomyocyte apoptosis, the overexpression of miR-182 protected cells from either Nogo-C or CoCl_2_-induced apoptosis, as assayed by flow cytometry (Figure 5g). Together, our results indicate that miR-182 has an important role in hypoxia-related cardiac diseases through negatively regulating Nogo-C expression.

## Discussion

Nogo family proteins are profoundly involved in multiple cellular processes. In addition to their well studied functions in nervous system, our present study found that Nogo-C is a determinant player in ischemia-related cardiomyocyte apoptosis during MI. There are several lines supporting our notions. First, Nogo-C protein is increased in MI heart and in hypoxic stimuli-induced apoptotic cardiomyocytes. Second, overexpression of Nogo-C *per se* is sufficient to induce cardiomyocyte apoptosis, whereas knockdown of Nogo-C prevents hypoxia-induced apoptosis. Third, *in vivo* knockout of Nogo-C (Nogo-C^−/−^) preserved cardiac function and protected cardiomyocytes against apoptosis after MI. Finally, we identified Nogo-C is a target gene of miR-182, which negatively regulated Nogo-C and is downregulated during MI.

Apoptosis is an important feature leading to the cardiac dysfunction after MI and ischemic heart diseases.^24, 25^ Thus, investigation of the molecular mechanisms underlying apoptosis is helpful for better understanding MI, and even for providing therapeutic targets for treatment of MI. In our current study, we found that both mRNA and protein levels of Nogo-C were upregulated in MI hearts, and the Nogo-C protein mainly increased in border area of infarct zone where most apoptotic cells enriched, suggesting a possible role of Nogo-C in the pathogenic process of cardiomyocyte apoptosis. Indeed, overexpression of Nogo-C in cardiomyocytes caused cell apoptosis, and knockdown of Nogo-C inhibited hypoxia-induced cardiomyocyte apoptosis. Our findings that Nogo-C regulates cell apoptosis in the heart are in general agreement with findings of Nogo-A, one of the splicing isoforms of Nogo-C, in cardiomyocytes.^23^ Although highly expressed in central nervous system, Nogo-A has also been reported to be upregulated in human hearts of dilated cardiomyopathy and rat neonatal cardiomyocytes subjected to hypoxia/reperfusion, and knockdown of Nogo-A inhibited hypoxia/reperfusion induced cardiomyocyte apoptosis through inhibition of mitochondria-related cell death pathway, ROS accumulation, and diastolic calcium abnormality.^23^ Structurally, Nogo-C shares the transmembrane domain and C-terminal domain with Nogo-A, whereas missing the N-terminal domain contained by Nogo-A protein.^26, 27^ So, it is possible that the common domains shared by the two Nogo proteins contribute to the apoptotic effects, the same as the inhibitory effect of regeneration in nervous system.^18^ However, the exact molecular mechanisms of Nogo-C-induced cardiomyocyte apoptosis need further exploration.

Our above results on Nogo-C and others' previous studies on Nogo-A indicate that the increased Nogo proteins during MI or ischemic heart diseases may contribute to cardiac dysfunction through regulating cardiomyocyte apoptosis. To better understand the pathophysiological significance of Nogo-C in the heart, we generated the Nogo-C knockout mouse model, the first Nogo-C knockout model to our knowledge. Our *in vivo* functional study in Nogo-C null mice provides solid evidence supporting our hypothesis, that depletion of Nogo-C protected cardiomyocyte apoptosis in MI heart, largely decreased IR, and most importantly, preserved cardiac function after MI injury, suggesting that Nogo-C may serve as a therapeutic target for treatment of MI or ischemia-related cardiac diseases. Our present study of the protective effect of Nogo-C knockout in the heart is in general agreement with Nogo-A knockout mouse model.^23^ Although Nogo-C knockout protected the heart from MI damage, it showed no cardiac phenotype at basal level, suggesting that Nogo-C is either dispensable for normal functions or there are redundant pathways in the heart. Our preliminary data found that mRNA levels of other two Nogo family members, Nogo-A and Nogo-B, were increased in Nogo-C KO mouse hearts, suggesting that elevated Nogo proteins may at least partially provide compensatory function in Nogo-C KO hearts. In this respect, the protective effect of Nogo-C knockout independent of the compensatorily increased Nogo-A suggests that Nogo-C and Nogo-A may differentially regulate cardiomyocyte apoptosis. Indeed, Nogo-A induced cardiomyocyte apoptosis through a mitochondria dependent pathway,^23^ whereas Nogo-C had no effect on mitochondria (data not shown).

MicroRNAs (miRNAs) have a crucial role in the pathogenesis of various cardiac diseases including MI, post-infarct fibrosis, and heart failure.^28, 29, 30, 31, 32, 33^ In mouse and human hearts, the expression profile of miRNAs in the border zone of infarct region was altered after MI and during the subsequent cardiac fibrosis.^32^ Downregulation of miRNAs especially miR-29 family de-repressed its target genes, which encode fibrosis-related proteins, causing post-infarct fibrosis and cardiac dysfunction.^34^ In the current study, we predicted, according to the 3′UTR region of Nogo-C, that Nogo-C is a target gene of miR-182. *In vitro* expression of miR-182 reduced Nogo-C protein level and Nogo-C 3′-UTR luciferase reporter activity, confirming our prediction that miR-182 negatively regulates Nogo-C expression. Moreover, the expression of miR-182 was decreased in the border zone of infarct heart and in hypoxic cardiomyocytes, suggesting that miR-182 may be involved in the pathological processes of MI and ischemic heart diseases. MiR-182 has been reported to participate in the regulation of multiple physiological and pathological processes including retinal development and degeneration,^35, 36^ cancers,^37, 38, 39, 40^ inflammation,^41, 42^ and cardiac hypertrophy.^43^ Controversially, miR-182 showed tumor-suppressive function in some cancers such as glioblastoma,^44^ whereas displayed ongogenic function in other cancers such as lung adenocarcinoma.^45^ In our study, we found that miR-182 protected cardiomyocytes from Nogo-C and hypoxia-induced apoptosis. It is unclear why miR-182 functions differentially on cell growth or apoptosis in cancers or in the heart, tissue specific effect might be a possible reason for this discrepancy. However, how miR-182 is regulated during MI and ischemic heart is not yet clear and merits further investigation.

In summary, our present study found that Nogo-C protein was upregulated in MI heart and hypoxic cardiomyocytes, the increased Nogo-C induced cardiomyocyte apoptosis and silencing Nogo-C protected cardiomyocytes from hypoxia-induced apoptosis. Knockout of Nogo-C protected the heart from MI injury and preserved cardiac function. Furthermore, we identified that miR-182 is a negative regulator of Nogo-C, and downregulation of miR-182 may contribute to the increased Nogo-C and cardiac dysfunction during MI. Our findings reveal a critical role of Nogo-C in the heart, and thus shed new light on the development of therapeutic targets for ischemia-related cardiac diseases.

## Materials and Methods

### Mouse MI model

All procedures of animal handling were approved by the Institutional Animal Care and Use Committee of Peking University Health Science Center. Mouse MI model was established with C57BL/6 male mice at age of 8–12-week as previously described.^46^ Mice were anesthetized with intraperitoneal injection of pentobarbital sodium (60 mg/kg). The fourth intercostals space over the left chest was exposed and the heart was rapidly squeezed out of the thoracic space, the LAD below the tip of the left auricle was tied with a 6 sterile silk suture. The sham subjects underwent same operation except that the LAD was not ligated. Twenty-four hours after the operation, echocardiography was performed with a Vevo 710 RMV-707B (VisualSonics, Toronto, Ontario, Canada), and hearts were collected and prepared for experiments.

### Generation of Nogo-C knockout mouse model

Nogo-C knockout mice (Nogo-C^−/−^) were generated by TALEN technique in C57BL/6 background. Briefly, 8 basepairs of exon 1c of Nogo gene, the specific exon for Nogo-C, were chopped to induce a frame-shift mutation, resulting in a truncated protein, which may be subject to non-sense mediated decay. Knockout of Nogo-C was confirmed by western blot and gene sequencing.

### Immunohistochemical staining assay

Heart samples were fixed with 4% paraformaldehyde and imbedded with paraffin wax. The sections were perfused in 3% H_2_O_2_ to clear endogenous peroxidase and microwaved in sodium citrate buffer (1 mM, pH 6). The slides were blocked in 1% BSA for 30 min at 37 °C and were then probed with anti-Nogo-C antibody (1:100) overnight at 4 °C and a horseradish peroxidase conjugated secondary antibody at 37 °C for 30 min.

### Isolation and culture of rat neonatal cardiomyocytes

Rat neonatal cardiomyocytes were isolated and cultured as described previously.^22^ Briefly, ventricles of Sprague–Dawley rats postnatal 1 to 2 days were digested in HBSS solution (KCl 0.4 g/l, KH_2_PO_4_ 0.06 g/l, NaHCO_3_ 0.35 g/l, NaCl:8 g/l, Na_2_HPO_4_ 12H_2_O 0.12 g/l, glucose 1 g/l, pH 7.4) containing 0.1% trypsin (Invitrogen, Carlsbad, CA, USA) and 0.05% type II collagenase (Worthington, Lakewood, CO, USA). Cells were pre-plated for 2 h and the supernatant containing purified cardiomyocytes was collected and re-plated and cultured for another 48–72 h before transfecting adenovirus.

### Construction of adenovirus

The full-length cDNA of Nogo-C was amplified by PCR with primers: 5′-CACCATGGACG GACAGAAGAAACA-3′ (forward) and 5′-TCAATCTGCTTTGCGCTTCAATCC-3′ (reverse), the amplified product was then inserted into pENTR/TEV/D-TOPO vector (Invitrogen). Newly constructed product was recombined with pAd/CMV/V5-DEST vector (Invitrogen). Target sequence for Nogo-C-shRNA was 5′-GGCAGATCGTGGCAAGAAA-3′. The Nogo-C-shRNA sequence was inserted into pENTR^TM^/U6 vector and then recombined with pBLOCK-iT^TM^6-DEST vector. Adenovirus was produced with Adenoviral Expression System (Invitrogen) and purified using Vivapure AdenoPACK20RT Kit (Sartorius, Göttingen, Germany).

### Immunoblotting

Protein was extracted from mouse myocardium or isolated cardiomyocytes with Roth lysis buffer (HEPES 50 mM, NaCl 150 mM, EDTA 5 mM, EGTA 5 mM, NaF 20 mM, Triton X-100 1%, pH 7.4) supplemented with 100X protease inhibitor cocktail (Sigma, Santa Clara, CA, USA). A total of 40–100 ug protein was then separated by SDS-PAGE, and transferred to PVDF membranes. Membranes were probed with indicated primary antibodies (Nogo-C or *α*-tubulin), and then incubated with the secondary antibodies (horseradish peroxidase-conjugated anti-rabbit IgG from ZSGB-BIO, Beijing, China). Immunoblots were evaluated using the Chemi Doc XRS+instrument (Bio-RAD, Hercules, CA, USA).

### MiRNA assay

MicroRNA assay was detected with stem loop primers purchased from Ribobio (Guangzhou RiboBio, Guangzhou, China). U6 small nucleolar RNA (Guangzhou RiboBio) was used for the normalization. The sequence of rno-mir-182 was 5′-ACGCGGGUCUAGCUGCCGGAGGCCUCCCACCGUUUUUGGCAAUGGUAGAACUCACACCGGUA-3′. Rno-mir-182 mimics (Guangzhou RiboBio) were transfected to rat neonatal cardiomyocytes with Lipofectamine 3000 (Invitrogen).

### Dual luciferase reporter assay

Nogo-C 3′-UTR sequences were amplified by PCR with primers: 5′-GCGCTCGAGAAAAGC CCCAAACAGAAGT-3′ (forward) and 5′-AATGCGGCCGCAAACACTAAACACAAACAT-3′ (reverse). PCR products were then constructed into pmiR-RB-REPORT plasmid (Guangzhou RiboBio) containing two reporters, a renilla luciferase (hRluc) for expression evaluation, and a firefly luciferase (hluc) as an internal control for amount correlation. Hela cells were co-transfected with 100 ng Nogo-C 3′UTR reporter plasmid and 2 *μ*g rno-miR-182 mimic or empty vector. Cells were collected 48 h after transfection and analyzed using a Dual Luciferase Reporter Assay System (Promega, Madison, WI, USA). Luciferase activity was examined by Veritas luminometer (Turner BioSystems, Sunnyvale, CA, USA). Renilla luciferase activity of each sample was normalized to firefly luciferase activity.

### Cell death assay by TUNEL staining and flow cytometry

Cell apoptosis was determined by TUNEL (terminal deoxyribonucleotidyl transferase (TDT)-mediated dUTP-digoxigenin nick end labeling) assay using *In situ* cell death detection kit (Roche, Indianapolis, IN, USA), or by fluorescein isothiocyanate-conjugated annexin V/propidium iodide double staining flow cytometry using Annexin-V-FITC Apoptosis Detection Kit (Dojindo Laboratories, Kumamoto-ken, Japan).

### Transmission electron microscopy analyses

Hearts were fixed with 2% glutaraldehydein sodium cacodylate buffer (0.1 M, pH 7.2) at 4 °C overnight and post-fixed for 1 h in 1% osmiumtetroxide. Digital images were acquired by a JEM-1230 High Contrast Transmission Electron Microscope and Soft Imaging system (JEOL, Tokyo, Japan).

### TTC staining

Mice were killed with anesthetic 24 h after MI operation. Hearts were frozen at −20 °C for 20 min, and then were sectioned into 5 mm thick slices. Five continuous slices from apex to the occlusion site were incubated in triphenyltetrazolium chloride (TTC) at 37 °C for 5 min. After fixation with 4% paraformaldehyde overnight, each slice was weighed (*w*) and photographed with a digital camera. Infarct areas were indicated as the area not stained by TTC. The IR and left ventricular size (LV) were evaluated by Photoshop. Percentage of infarct area was calculated as IR/LV=(∑IR_(per slice)_ × *w*_(per slice)_)/(∑LV_(per slice)_ × *w*_(per slice)_).

### LDH assay

One milliliter vein blood was collected from right ventricular with a syringe and then centrifuged at 3000 r.p.m. for 15 min, 4 °C. Serum samples were sent to clinical laboratory of the Third Hospital at Peking University for analyses of LDH levels.

### Statistical analysis

Data are presented as mean±S.E.M. Statistical significance of differences between groups was analyzed by unpaired *t*-test or one-way ANOVA followed by SNK (Student–Newman–Keuls) when more than two groups were compared. *P*<0.05 was considered statistically significant.

## Figures and Tables

**Table 1:** Echocardiographic assay of cardiac function in wildtype control and Nogo-C knockout mice

Echocardiographic parameter	WT (*n*=7)	Nogo-C^−/−^ (*n*=6)
	Mean±S.E.M.	Mean±S.E.M.
LVID-d (mm)	3.221±0.0649	3.417±0.1072
LVPW-d (mm)	0.7886±0.07595	0.7633±0.06647
LVID-s (mm)	2.119±0.107	2.287±0.08686
LVPW-s (mm)	1.169±0.09277	1.13±0.07014
EF(%)	64.18±3.544	62.43±2.244
FS(%)	34.38±2.643	33±1.575

Abbreviations: LVID-d, left ventricular internal diameter at end-diastole; LVPW-d, left ventricular posterior wall thickness at end-diastole; LVID-s, left ventricular internal diameter at end-systole; LVPW-s, left ventricular posterior wall thickness at end-systole; EF%, ejection fraction; FS%, fractional shortening

## Abbreviations

MI: myocardial infarction
ROS: reactive oxygen species
3′UTR: 3′ untranslated regions
TUNEL: terminal deoxyribonucleotidyl transferase (TDT)-mediated dUTP-digoxigenin nick end labeling
AV: annexin V
TTC: triphenyltetrazolium chloride
IR: infarct size
LV: left ventricule
LAD: lactate dehydrogenase
shRNA: short hairpin RNA
miRNA: microRNA
LVID: left ventricular internal diameter
LVPW: left ventricular posterior wall thickness
EF: ejection fraction
FAC: fractional area change
HW/BW: heart weight to body weight ratio
HW/TL: heart weight to tibia length ratio
HE: hematoxylin/eosin
